# Emergency Medical Services Capacity for Prehospital Stroke Care in North Carolina

**DOI:** 10.5888/pcd10.130035

**Published:** 2013-09-05

**Authors:** Mehul D. Patel, Jane H. Brice, Kelly R. Evenson, Kathryn M. Rose, Chirayath M. Suchindran, Wayne D. Rosamond

**Affiliations:** Author Affiliations: Jane H. Brice, Kelly R. Evenson, Chirayath M. Suchindran, Wayne D. Rosamond, University of North Carolina at Chapel Hill, Chapel Hill, North Carolina; Kathryn M. Rose, SRA International, Durham, North Carolina.

## Abstract

**Introduction:**

Prior assessments of emergency medical services (EMS) stroke capacity found deficiencies in education and training, use of protocols and screening tools, and planning for the transport of patients. A 2001 survey of North Carolina EMS providers found many EMS systems lacked basic stroke services. Recent statewide efforts have sought to standardize and improve prehospital stroke care. The objective of this study was to assess EMS stroke care capacity in North Carolina and evaluate statewide changes since 2001.

**Methods:**

In June 2012, we conducted a web-based survey on stroke education and training and stroke care practices and policies among all EMS systems in North Carolina. We used the McNemar test to assess changes from 2001 to 2012.

**Results:**

Of 100 EMS systems in North Carolina, 98 responded to our survey. Most systems reported providing stroke education and training (95%) to EMS personnel, using a validated stroke scale or screening tool (96%), and having a hospital prenotification policy (98%). Many were suboptimal in covering basic stroke educational topics (71%), always communicating stroke screen results to the destination hospital (46%), and always using a written destination plan (49%). Among 70 EMS systems for which we had data for 2001 and 2012, we observed significant improvements in education on stroke scales or screening tools (61% to 93%, *P* < .001) and use of validated stroke scales or screening tools (23% to 96%, *P* < .001).

**Conclusion:**

Major improvements in EMS stroke care, especially in prehospital stroke screening, have occurred in North Carolina in the past decade, whereas other practices and policies, including use of destination plans, remain in need of improvement.

## Introduction

Emergency medical services (EMS) can have a positive impact on the care of acute stroke patients through early identification and expedited transport and thus more timely delivery of treatments, notably thrombolytic therapy (1). With proper education and use of protocols, EMS personnel can screen for stroke in the field, initiate patient evaluation, and transport patients to a specialized stroke center (2–4). However, current levels of EMS education and prehospital care practices for stroke patients are not well characterized and vary by location (5–7).

Improving EMS capabilities to respond to and manage care of acute stroke patients is important because stroke is a major cause of death and disability in the United States (8). In North Carolina, state legislation was passed in 2006 to address the availability of stroke-related resources among hospitals and EMS systems (9). This legislation led to the development and implementation of standardized EMS stroke care practices and policies. By 2010, all EMS systems in North Carolina were required to use a standardized protocol to guide the prehospital care of stroke patients and a written destination plan to facilitate the transport of stroke patients to the most appropriate hospital.

A statewide survey in 2001 of EMS stroke care in North Carolina found EMS education and the use of protocols to be lacking (5). However, in the past 10 years, major national and statewide changes have occurred in the prehospital management and care of stroke patients, including the use of standardized protocols and validated stroke screening tools and the development and use of destination plans (10–12).

The objective of this study was to examine current EMS stroke education and stroke care practices and policies in North Carolina and to evaluate statewide changes since 2001. Given advancements in prehospital stroke care and recent EMS implementation of stroke policies, we hypothesized improvements in EMS stroke care capacity during the past decade.

## Methods

### Study design and data collection

We developed a 31-item web-based survey to collect information on the stroke care capacity of EMS systems in North Carolina. The survey measured frequency and educational content of stroke training sessions and information about stroke care practices and policies. Questions were adopted from other published surveys of EMS stroke care capacity (5,6) or developed on the basis of expert input from 2 local EMS medical directors. We also assessed general EMS system characteristics, including pay structure and level of service. The survey instrument and methodology were approved by the institutional review board of the University of North Carolina at Chapel Hill. A copy of the survey is available at www.unc.edu/~kevenson/_2012_NC_EMS_StrokeSurvey.pdf.

The 100 county-based EMS systems in North Carolina comprise approximately 35,000 EMS personnel and 540 EMS agencies (13). We identified 100 EMS administrative directors through the state regulatory office directory and invited them to complete the web-based survey. We selected these directors as key informants because they supervise EMS personnel and manage the daily operations of their systems. Survey instructions encouraged respondents to elicit information from others in their organizations, such as training officers and medical directors. Links to the online survey were e-mailed in June 2012. We sent reminder e-mails 1 and 2 weeks after the initial invitation. We also made follow-up telephone calls to nonrespondents and offered the option to complete the survey by telephone.

### Data processing and analysis

We devised a summary score of EMS stroke care capacity using parameters recommended by national and local experts (10,12). Ideally, a stroke-capable EMS system should address 4 priority areas: education and training, protocol and screening, destination plan, and continuous quality improvement. Each EMS system responding to our survey was assessed for each priority area and assigned points according to our assessment. Each area was equally weighted with a maximum of 3 points, thus allowing an overall maximum stroke care capacity score of 12 points (Box).

Box. Emergency Medical Services (EMS) Stroke Care Capacity Scoring System, North Carolina, 2012

**Table:** 

Priority Areas and Measures	Points
**Education and training**	
At least 2 hours of stroke training provided per year	1
Personnel trained on stroke at least once per year	1
Training covers basic stroke educational topics^a^	1
**Protocol and screening**	
Standardized stroke protocol	1
Validated stroke scale or screening tool^b^	1
Always communicate stroke scale or screen results to hospital	1
**Destination plan**	
Written stroke destination plan	1
Always use the stroke destination plan	1
Plan to transport to a stroke center	1
**Continuous quality improvement^c^**	
Data-driven performance feedback on stroke care in past year	3
**Maximum EMS stroke-care capacity score**	12

a Basic topics were stroke risk factors, signs and symptoms; pathophysiology; and scale or screening tool.

b Validated stroke scales and screens used by survey respondents were the Los Angeles Prehospital Stroke Screen (3), the Cincinnati Prehospital Stroke Scale (2), and the Miami Emergency Neurologic Deficit examination (14).

c Systems were characterized as engaging in continuous quality improvement if they examined standard electronic data in the past year to evaluate their stroke care (15).

We calculated descriptive statistics for the scores among all responding systems. Overall scores were categorized into 4 groups: 0 to 3 points, 4 to 6 points, 7 to 9 points, and 10 to 12 points. We compared frequencies of scores by estimated annual patient volume of the EMS system and by county population density. We estimated annual patient volume as the number of EMS events in the past year, as recorded in the North Carolina Credentialing Information System (16), and then categorized patient volume into 3 groups: fewer than 5,000 events, 5,000 to 20,000 events, and more than 20,000 events. County population density was categorized as metropolitan, micropolitan, or rural as defined by the US Office of Management and Budget (17).

In 2001, a survey mailed to 83 EMS agencies in North Carolina was completed and returned by 72 of them (5). To enable comparison between the 2012 and 2001 surveys, we repeated questions on stroke education, transport with lights and sirens on, use of validated stroke screening tools, and policy on advance notification of hospitals. We acquired the 2001 survey responses from the study authors and matched them by EMS system to our survey responses. The comparison analysis was restricted to the 70 EMS systems for which we had data for both years. We compared this subset of 70 EMS systems with all EMS systems in North Carolina by patient volume, number of EMS personnel, and level of service and found minimal differences.

We calculated both absolute and relative changes in EMS stroke care capacity measures between 2001 and 2012. A relative change greater than 10% was considered meaningful. We tested the difference between paired proportions by using the 2-sided McNemar exact test. The 2-sided Fisher exact test was used for categorical data and the Wilcoxon rank sum test for non-normal continuous data. A *P* value less than .05 was considered significant.

## Results

### North Carolina EMS systems

Of 100 EMS systems in North Carolina, 2 systems provided basic life-support service only, and 98 systems provided all or some advanced life support service. The EMS systems varied by number of certified EMS personnel (median, 120 personnel; interquartile range [IQR], 66–235 personnel) and by estimated annual patient volume (median, 8,004 patients; IQR, 3,754–17,848 patients) (16). Based on county population estimates, 40 systems serviced metropolitan areas, 30 micropolitan, and 30 rural.

### 2012 EMS stroke survey

We received survey responses from 98 of 100 EMS systems. Most respondents completed the survey online; 9 surveys were conducted by telephone. Primary survey respondents were administrative directors (n = 80), training officers (n = 12), and 1 medical director; 5 respondents did not report their job title. Seven surveys had 2 respondents (eg, a director and a training officer, an administrative director and an emergency department nurse). 

Most systems (95%) provided at least 1 stroke training session to EMS personnel in the past 2 years (Table 1); of these, 74% provided stroke education at least once per year. The educational content of training sessions always included stroke signs and symptoms and frequently included stroke scales or screening tools (95%); thrombolytic therapy was addressed less frequently (66%). In-person classroom training sessions were almost always offered, but online courses and videos were also used. Almost all surveyed EMS systems used a validated stroke scale or screening tool, such as the Los Angeles Prehospital Stroke Screen (66%) (3) or the Cincinnati Prehospital Stroke Scale (52%) (2). However, only 46% reported always communicating stroke scale or screen results to the destination hospital. Similarly, only 49% reported always using a written plan to determine the destination hospital. Lastly, 98% of EMS systems reported having a policy to notify the hospital in advance when transporting a suspected stroke patient.

**Table 1:** Characteristics of Emergency Medical Services (EMS) Stroke-Care Capacity Among 98 EMS Systems, North Carolina, 2012

Domains and Measures	Survey Results^a^
**Education and Training**	
Stroke training provided in past 2 years	93 (95)
Median (IQR) hours of stroke training provided in past 2 years^b^	7.0 (4.0–10.0)
**Frequency of stroke training^b^**	
More than once per year	21 (23)
Once per year	47 (51)
Every 2 or more years	21 (23)
Only when initially certified	3 (3)
**Stroke educational topics covered in training^b^^,^ c**	
Risk factors	74 (80)
Signs and symptoms	92 (100)
Pathophysiology	72 (78)
Scale or screening tool	87 (95)
Thrombolytic therapy	61 (66)
All 5 stroke educational topics covered^b^	50 (54)
**Format of stroke training sessions^b^^,^ c**	
Classroom	91 (99)
Online	41 (45)
DVD or video	21 (23)
**Practices and Policies**	
**Lights and sirens are used while transporting suspected stroke patients**	
Yes	30 (31)
No	10 (10)
Choice made by crew	58 (59)
**Validated stroke scale or screening tool is used^d^**	94 (96)
**Stroke scale or screening tool identified as being used^c^^,^ e**	
Los Angeles Prehospital Stroke Screen	62 (66)
Cincinnati Prehospital Stroke Scale	49 (52)
Miami Emergency Neurologic Deficit examination	17 (18)
**Frequency of communicating stroke scale or screen results to destination hospital^e^**	
Always	43 (46)
Very often	44 (47)
Sometimes	5 (5)
Rarely	2 (2)
Never	0
**Frequency of using a written plan to determine destination hospital^f^**	
Always	47 (49)
Very often	37 (39)
Sometimes	6 (6)
Rarely	5 (5)
Never	1 (1)
Policy exists to notify hospital in advance if stroke suspected	96 (98)

a All values are numbers (percentages) unless otherwise indicated.

b Of the 93 EMS systems that provided stroke training in past 2 years; 1 system did not answer this question.

c Categories are not exclusive.

d Validated stroke scales and screens identified in the survey were the Los Angeles Prehospital Stroke Screen (3), the Cincinnati Prehospital Stroke Scale (2), and the Miami Emergency Neurologic Deficit examination (14).

e Among the 94 EMS systems that used a validated stroke scale or screen.

f Two systems did not answer this question.

### EMS stroke care capacity score

**Figure 1:**
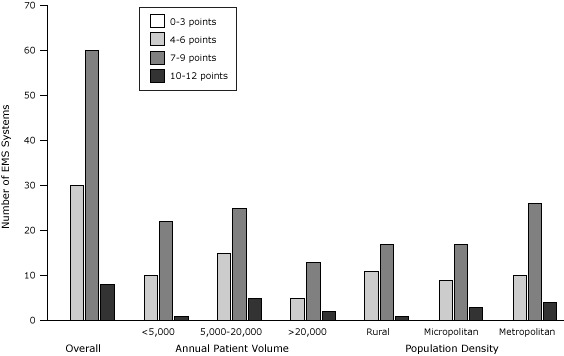
Emergency Medical Services (EMS) stroke care capacity scores for 98 EMS systems responding to survey, overall and by patient volume and county population density, North Carolina, 2012. No system scored 0 to 3 points. County population density was categorized as metropolitan, micropolitan, and rural as defined by the US Office of Management and Budget (17). **Table:** 

Among the 98 EMS systems analyzed, stroke care capacity scores ranged from 4 to 12 points. The median score was 7 points (IQR, 6–9 points), and 3 systems scored the maximum 12 points. Most systems provided at least 2 hours of stroke training per year (78%), educated personnel at least once per year (69%), and covered the basic stroke educational topics (66%); only 44% did all 3 of these activities, and 12% did none. Only 44% of systems scored the maximum 3 points for protocol and screening, and only 45% scored the maximum 3 points for destination plan, even though the state regulatory office requires each system to implement a standardized stroke protocol and written destination plan. Performance feedback was uncommon: only 13% of systems had evaluated their stroke patient care data in the past year. Overall, EMS stroke care capacity scores in North Carolina showed room for improvement (Figure 1). Although no EMS system scored fewer than 4 points overall, 30 systems scored 6 points or fewer. We observed high scores (ie, 10–12 points) in all categories of patient volume and county population density.

	Stroke Care Capacity Scores			
	0–3 Points	4–6 Points	7–9 Points	10–12 Points
**Overall, no. of systems**	0	30	60	8
**Estimated annual patient volume, no. of systems**				
<5,000	0	10	22	1
5,000–20,000	0	15	25	5
>20,000	0	5	13	2
**County population density,^a^ no. of systems**				
Rural	0	11	17	1
Micropolitan	0	9	17	3
Metropolitan	0	10	26	4

^a^ County population density was categorized as metropolitan, micropolitan, and rural as defined by the US Office of Management and Budget (17).

### Comparison between 2001 survey and 2012 survey

**Figure 2:**
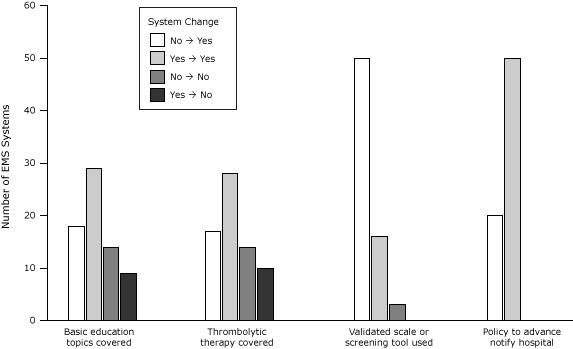
Changes in selected stroke care capacity measures from 2001 to 2012 among 70 Emergency Medical Services systems, North Carolina. The 70 systems participated in surveys administered in 2001 and 2012. Basic education topics were stroke risk factors, signs and symptoms, pathophysiology, and scale or screening tool. Not included in basic topics was thrombolytic therapy. One system did not answer the question on thrombolytic therapy, and one did not answer the question on use of validated scale or screening tool. **Table:** 

We observed a moderate increase in the percentage of EMS systems providing stroke education and in the overall median number of hours of stroke education provided (Table 2). Although the percentage of systems providing education on stroke risk factors and pathophysiology decreased, the percentage providing education on stroke signs and symptoms and thrombolytic therapy increased. We observed a significant increase (31 percentage points, absolute; 51%, relative; McNemar exact test = 18.6, *P* < .001) in the percentage of systems providing education on stroke scales and screening tools. Coverage of the basic 4 stroke educational topics also increased from 54% to 67%. The greatest change was the increase in use of validated stroke scales or screening tools (from 23% to 96%; McNemar exact test = 50.0, *P* < .001). A policy to notify hospitals in advance of stroke patient arrival existed at 71% of systems in 2001; all systems had adopted this policy by 2012. Although18 systems changed from not covering all 4 basic educational topics to covering them and 9 changed from covering to not covering them (Figure 2), the result was a 13 percentage-point increase in the proportion of systems covering these topics. The 72 percentage-point increase in the use of validated stroke scales or screening tools was solely driven by 50 systems that changed positively.

**Table 2:** Changes in Emergency Medical Services (EMS) Stroke-Care Capacity by 70 EMS Systems From 2001 to 2012, North Carolina^a^

Domains and Measures	2001 Survey, %	2012 Survey, %	Absolute Change, Percentage Points	Relative Change, %	*P *Value^b^
**Education and Training**					
Stroke training provided in past 2 years	90	97	7	8	.18
Median no. of hours of stroke training provided in past 2 years^c^	4.0	6.0	2.0	50	.08^d^
**Stroke topics covered in training^c^**					
Risk factors	81	77	−4	−5	.70
Signs and symptoms	89	97	9	10	.11
Pathophysiology	81	74	−7	−9	.36
Scale or screening tool	61	93	31	51	<.001
Thrombolytic therapy	55	65	10	18	.25
4 Basic stroke educational topics covered^c^^,^ e	54	67	13	24	.12
**Practices and Policies**					
**Suspected stroke patients transported with lights and sirens on, %**					
Yes	11	31	NA		.85^f^
No	17	9			
Choice made by crew	71	60			
**Validated stroke scale or screening tool used^g^**					
Yes	23	96	72	312	<.001
**Policy to notify hospital in advance if stroke suspected**					
Yes	71	100	29	40	—h

Abbreviations: NA, not applicable.

a Although 98 EMS systems responded to the 2012 survey, we had 2001 survey data (5) for only 70 systems. Units indicated in column headings apply to all data in column, except for data on number of hours of stroke training.

b Determined by 2-sided McNemar exact test unless otherwise indicated.

c Systems not providing stroke training were recorded as 0 hours of training provided and no educational topics covered.

d Determined by Wilcoxon rank sum test.

e Basic topics were stroke risk factors, signs and symptoms, pathophysiology, and scale or screening tool. Not included was thrombolytic therapy.

f Determined by Fisher exact test.

g Validated stroke scales and screens named on the survey were the Los Angeles Prehospital Stroke Screen (3), the Cincinnati Prehospital Stroke Scale (2), and the Miami Emergency Neurologic Deficit examination (14).

h Statistic not computed because 2012 data had only 1 response level.

Survey Measure	EMS System Changes From 2001 to 2012			
	Changed from No to Yes	Stayed at Yes	Stayed at No	Changed from Yes to No
4 Basic educational topics were covered^a^	18	29	14	9
Thrombolytic therapy was covered as an educational topic^b^	17	28	14	10
Validated scale or screening tool was used^b^	50	16	3	0
Policy to advance notify hospital existed	20	50	0	0

^a^ Basic education topics were stroke risk factors, signs and symptoms, pathophysiology, and scale or screening tool. Not included in basic topics was thrombolytic therapy.

^b^ One system did not answer.

## Discussion

Our study found that some aspects of EMS stroke care in North Carolina were practiced almost universally, including stroke education, use of validated stroke scales or screening tools, and a policy to notify hospitals in advance of suspected stroke. However, data on other measures of stroke education and prehospital practice and policies suggested room for improvement. Among EMS systems that provided stroke training sessions, almost one-third did not cover the basic stroke educational topics. Of the systems surveyed, 69% educated their personnel on stroke at least annually. This percentage was only moderately greater than the 60% found in a study of EMS agencies in Minnesota in 2006, one of the few published, statewide assessments of EMS stroke care capacity (6).

Although almost all EMS systems in North Carolina used a validated stroke scale or screening tool, fewer than half regularly communicated the results to the destination hospital. This finding is consistent with the finding that only 34% of EMS agencies in Minnesota verbally communicated stroke scale findings (6). Moreover, almost all (98%) systems in North Carolina reported a policy to notify hospitals in advance of suspected stroke patients. There appears to be an inconsistency between policy and compliance. Previous studies observed that prenotification by EMS personnel of a suspected stroke can significantly reduce in-hospital delays and increase treatment rates (18–20). Follow-up to our quantitative work could use qualitative methods or intervention studies to better understand the translation of advance notification policies into EMS communication practices.

Bypassing local community hospitals for specialized stroke centers by EMS is a recommended policy and practice for many stroke systems of care (10). Furthermore, all EMS systems in North Carolina are required to implement a destination plan for stroke. In comparison, only 37% of EMS agencies in Minnesota reported having such a plan (6). Although a plan is required in North Carolina, our survey showed that only about half of EMS systems always used their plan and another 12% never or only sometimes used it, suggesting that even with a statewide policy, local systems are complying at varying degrees. Differences in publicizing legislation and enforcing EMS policies across the state may have had an impact on local compliance, although we did not investigate these differences.

We found room for overall improvement in EMS stroke care capacity: 92% of systems scored less than 10 points of a possible 12. Of the main priority areas, continuous quality improvement was the least frequently addressed; only 13 systems (13%) had examined stroke care performance data in the previous year. A Utah-based study examined the feasibility of using electronic EMS records for monitoring prehospital stroke care and found that only 58% of EMS agencies entered data into an electronic system and that data were often incomplete (21). EMS systems in North Carolina are required to enter standardized data elements electronically, so all systems should have the necessary data for performance feedback (12). Moreover, a statistical analysis report on stroke patient data was recently designed and developed to improve EMS systems (12). Nonetheless, we found few systems generate these reports, and more work is needed to encourage data-driven continuous quality improvement in North Carolina and in other states.

Although low patient volume and rural locations are reported to limit EMS stroke care capacity (7,22–25), our comparisons by patient volume and county population density did not demonstrate strong variation by these characteristics. In fact, our findings show that some systems with low patient volume in rural areas have high stroke care capacity. However, further investigation using a larger sample size is needed to examine the relationship between EMS stroke care capacity and patient volume and county population density.

EMS capacity in North Carolina improved since 2001, especially in education and use of a validated stroke scale or screening tools, such as the Los Angeles Prehospital Stroke Screen or the Cincinnati Prehospital Stroke Screen. Our findings are consistent with those of similar studies. In 2006, 47% of Minnesota EMS agencies reported using the Cincinnati Prehospital Stroke Screen (6), whereas a more recent study (7) found that 80% of EMS agencies in 9 states reported using a stroke scale (although the scale used was not identified). Other significant positive changes were in education on all basic stroke topics, education on thrombolytic therapy, and a policy to notify hospitals in advance. Although we observed positive changes overall, we also found that certain EMS systems underwent negative changes (eg, in basic stroke educational topics). Although a formal policy evaluation was beyond the scope of this study, our findings show that statewide standardization of EMS stroke care was associated with improvements in capacity. Other states and regions that implement similar policies may also undergo significant improvements.

Our study has several limitations. Although our survey questions were not validated, they are similar to those in other surveys and were developed with input from subject matter experts (5,6). Our results were based on self-reports and are subject to inaccurate responses. However, respondents were selected because of their knowledge of their systems; in addition, respondents could work with others in completing the survey. Only 2 of the 100 systems did not respond, and systems that did not participate were well represented by the survey respondents according to service level (ie, basic life support, advanced life support), patient volume, and county population density. Our EMS stroke care capacity score was based on expert opinion and guideline recommendations but has not been independently validated. However, it can be easily replicated in other regions, and we believe it provides a useful summary of overall EMS capacity for stroke. In the absence of guidance in published literature, we chose to weight each priority area equally. We encourage further research to improve on this scoring method. A strength of our study was the direct system-specific comparisons between 2 years of data. Although we compared changes in 70 EMS systems only, these systems serviced about 81% of the 9.5 million residents in North Carolina. This subset of systems was similar to all EMS systems in North Carolina in level of service, patient volume, and county population density.

Our findings demonstrate areas of progress and areas that need improvement if EMS systems are to provide the best care for stroke patients in North Carolina. Education of EMS personnel should continue to be a focus, especially the content of stroke training sessions. Significant progress occurred in the institution of standardized patient care protocols, validated scales and screening tools, destination plans, and advance notification policies. However, the use of destination plans and communication to hospitals of stroke screen results need improvement. Given its large stroke burden and recent statewide actions to advance stroke care, North Carolina was a unique setting for this study. Many of the improvements observed in this study could be explained by statewide efforts to standardize prehospital stroke care and encourage best practices, such as bypassing local hospitals for stroke centers, although secular trends also likely played a role. Although other states may not require standardized protocols and destination plans, this study offers an example of how stroke care capacity can improve after such a policy is put in place. For local health services planning and quality improvement, it is important to continuously monitor the capacity of EMS systems to respond to and manage stroke patients. Further study is needed to understand how stroke capacity translates into actual EMS care received.
